# Pectin from Citrus Canning Wastewater as Potential Fat Replacer in Ice Cream

**DOI:** 10.3390/molecules23040925

**Published:** 2018-04-17

**Authors:** Hua Zhang, Jianle Chen, Junhui Li, Chaoyang Wei, Xingqian Ye, John Shi, Shiguo Chen

**Affiliations:** 1 Zhejiang Key Laboratory for Agro-Food Processing, Department of Food Science and Nutrition, Fuli Institute of Food Science, Zhejiang University, Hangzhou 310058, China; 21613072@zju.edu.cn (H.Z.); 3090100118@zju.edu.cn (J.C.); 21413027@zju.edu.cn (J.L.); 11513040@zju.edu.cn (C.W.); psu@zju.edu.cn (X.Y.); 2 Guelph Food Research Center, Agriculture and Agri-Food Canada, Guelph, ON N1G 5C9, Canada; john.shi@agr.gc.ca

**Keywords:** citrus canning wastewater, fat replacer, pectin, ice cream

## Abstract

Pectin had been recovered from canning wastewater produced by chemical treatment of segment membrane during preparation of canned citrus in our previous research. The purpose of this study was to characterize the extracted pectin from canning wastewater, and to evaluate its application as a fat alternative to replace fat in ice cream. The monosaccharide composition and rheological properties of the pectin were determined. The influences of fat reduction and pectin addition on the physicochemical, rheological and sensory properties of low-fat ice cream were determined. The rheological results showed that pectin solutions were typical pseudoplastic fluids. The addition of pectin in ice cream can cause an increase in viscosity, overrun, and hardness, and a decrease in meltdown of the ice cream. When 0.72% pectin (*w*/*w*) is incorporated into ice cream, a prototype product of ice cream with 45% lower fat content compared to the control was made. Results indicated that their qualities such as appearance, flavor, and taste were not significantly different. The low-fat ice cream had higher smoothness scores and lower mouth-coating scores. Hence, pectin extracted from citrus canning wastewater can be potentially used as fat replacer in ice cream, which benefits both the environment and the food industry.

## 1. Introduction

Canned citrus segments are popular among consumers worldwide with a big global market. China is one of the largest citrus-producing countries in the world and provides nearly 70% canned citrus segments for the international market [1]. Among traditional citrus canning, chemical treatments are essential procedures to remove membrane segments, resulting in a large amount of wastewater—about 600 m^3^ per day—from a single median-size factory among the nearly 100 factories in China [2]. The wastewater, namely segment membrane solution, contains high amounts of organic substances (pectin principally), resulting in a very high chemical oxygen demand (COD), and an environment challenge. 

Pectin is a heteropolysaccharide block co-polymer comprising 1,4-α-linked galacturonic acid and 1,2-linked rhamnose with side chains of either 1,4-linked β-d-galactose or 1,5-α-linked l-arabinose. Some of the C-6 carboxyl units of the galacturonic acid backbone are esterified with methoxyl groups or exist as uronic acid salt [3]. Pectin is approved worldwide for use in foods as a kind of safe food additive. It has also been reported to have many beneficial health effects, such as anti-inflammatory, anti-cancer, and hypocholesterolemic activities [4]. Due to its strong gelling and thickening properties, pectin has been used extensively in a variety of foods to control their texture and rheology. Now pectin is widely used as a fat alternative to replace fat in several food products because it possesses similar textural properties and mouthfeel to fat. It was reported that water-soluble pectin-enriched materials were extracted from apple pomace and were used to replace shortening in cookie formulations [5]. In the research conducted by Lim et al. [4], the pectin extracted from Yuja pomace was incorporated into cake formulations as a fat replacer to evaluate the baking properties of cakes. However, the supply of pectin in the global market is still short, although its annual trade value surpassed $850 million in 2013, and still rises steadily [3]. Finding new sources of pectin has always been a focus of research [6,7,8]. 

Ice cream has been identified as a three-phase made up of a network of fat globules and ice crystals dispersed in a high-viscosity aqueous phase [9]. As one of the main popular frozen desserts, it is generally regarded as a high-fat (10–16%) and high-caloric food. However, consumers are becoming more interested in low-fat food as they become concerned by the high risk from fat contents that are likely associated with cardiovascular diseases, hypertension and obesity [10]. Therefore, the dairy industry has studied and is looking for a way to develop low-fat and fat-free ice cream products. Compared with traditional ice cream, the low-fat ice cream has quality issues, such as low flavor and low textural qualities [11]. Thus, ice cream manufactures have high demand for milk fat replacers to produce more palatable products. The pectins are carbohydrate-based, due to their capability of interacting with water and thickening, gelling and emulsifying properties, and they can emulate the mouthfeel and flow properties of fat globules in aqueous systems [12]. 

In our previous study, a pilot plant-scale system has been established to recover pectin from processing wastewater [2]. Therefore, the aim of the present study was to determine and further investigate the rheological properties of the pectin, and to evaluate the effects of pectins as fat replacer on the physicochemical, rheological, and sensory properties of low-fat ice cream.

## 2. Results and Discussion 

### 2.1. Composition of Pectin 

Considering the high yield of pectin and the maximum utilization of both acid and alkaline wastewater from citrus canning, the pH of their mixtures was adjusted to the ranges of 3 to 4 before precipitation. The monosaccharide composition of pectin was determined. The pectin has a GalA content of 42.5 mol %, which was lower than the normal value of commercial pectin (> 65%). This most likely is due to the low extraction temperature [13]. Rhamnose (5.5 mol %), arabinose (18.6 mol %) and galactose (13.6 mol %) were the building units in both RG-I and RG-II. In addition, there was 17.3 mol % fucose. The presence of the trace amount of glucose (1.6 mol %) and mannose (0.7 mol %) can be regarded as the hydrolysis products of the residual cellulose and semi-cellulose. Other parameters such as esterification degree (48.84 ± 0.92%) uronic acid content (39.01 ± 0.23%) and molecular weight (537.7 ± 0.6%) have been published in our previous research [2]. The moisture content (3.9 ± 0.2%), acid insoluble ash (2.3 ± 0.3%), proteins content (0.9 ± 0.2%) and lipids (0.3 ± 0.1%) are tested according to AOAC.

### 2.2. Rheological Properties of Pectin 

**Figure 1:**
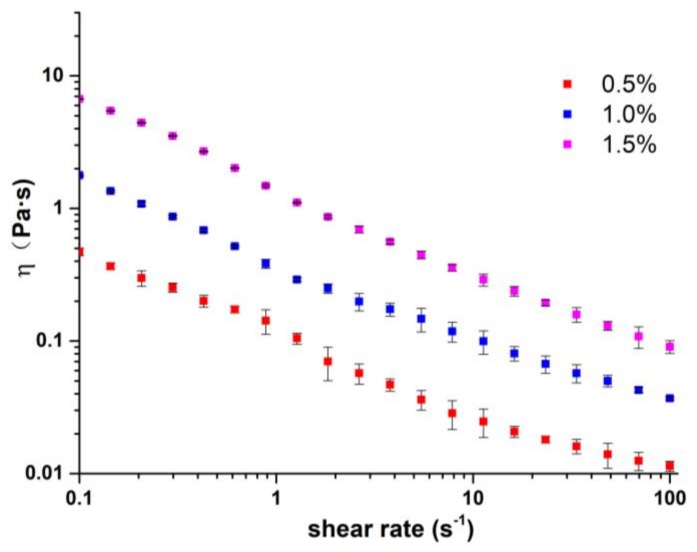
Rheological curves of pectin at different concentrations.

The rheological curves of pectin at different concentrations are shown in Figure 1. All solutions showed obvious shear thinning phenomena and behaved as pseudoplastic fluids. The pseudoplastic property was important for pectin in providing good sensory qualities, such as mouthfeel and flavor release and suspension properties of food products [14]. The shear thinning phenomenon was mainly related to the orientation of macromolecules along the direction of flow, and to the disentanglement of polysaccharides with increasing shear force [15]. In addition, this behavior can be characterized by the power law model (Equation (1)) [16].

(1) $$\eta =K\gamma^{\left(n-1\right)}$$

In Equation (1), where *η* is the apparent viscosity (Pa·s), K (Pa·s^n^) is the consistency index, *γ* is the shear rate (s^−1^) and *n* (dimensionless) is the flow behavior index. The fit parameters obtained are summarized in Table 1. In this model, the value of K can reflect the viscosity and flow resistance. The viscosity of the pectin solutions increased with increment of pectin concentration, which is probably due to the enhancement of the macromolecular entanglements [17]. The smaller the fluid characteristics index (*n*), the more obvious the polymer pseudoplasticity. As shown in Table 1, the *n* values decreased when the pectin concentrations increased, indicating the increased intertwining and breaking of polymer interactions. 

### 2.3. Rheological Properties of the Ice Cream Mix

**Figure 2:**
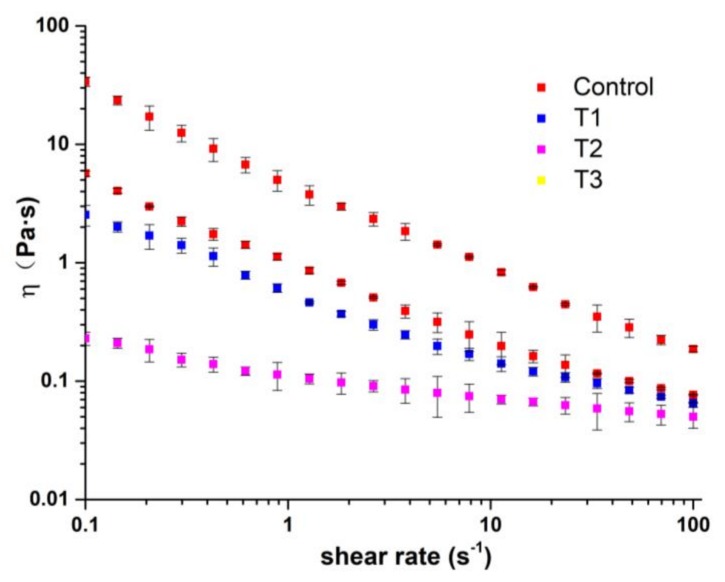
Rheological curves of different ice cream mixes.

The flow characteristics of ice cream slurry have great influence on the hardness, meltdown and overrun of ice cream products. Slurry with high viscosity can lead to difficult whipping, while too low viscosity of ice cream slurry produces air bubbles with poor stability that significantly affect quality and structure of finished ice cream products [18]. The rheological curves of ice cream mixes are shown in Figure 2. The parameters obtained by fitting to power-law model are shown in Table 2. All samples presented shear-thinning behavior and fitted the power-law model well (*R*^2^ ≥ 0.97). The results showed that the incorporation of pectin significantly changed the rheological properties of ice cream mixes, strengthened the viscosity development, and enhanced the shear thinning behavior as depicted by the increase of K values and the decrease of *n* value, respectively. Adding hydrocolloids capable of absorbing water and forming a gel-like network in addition to the other components of the mixes would obviously modify its rheology [19]. In the study by Schmidt et al. [11] ice cream made with a carbohydrate-based fat mimicker were also found to have greater viscosity, consistency coefficient, and smaller flow behavior index than those of the control. The K value increased from 0.12 to 5.06 Pa·s with the addition of pectin, the results are similar with a previous study in which K-values for ice cream mixes ranged from 0.17 to 3.67 Pa·s [20]. The range of *n*-values in this study (0.25–0.79) was wider than a previous study (0.36–0.50) in which various fat replacers were studied in low-fat ice cream [21]. 

### 2.4. Physicochemical Properties of Ice Cream

**Figure 3:**
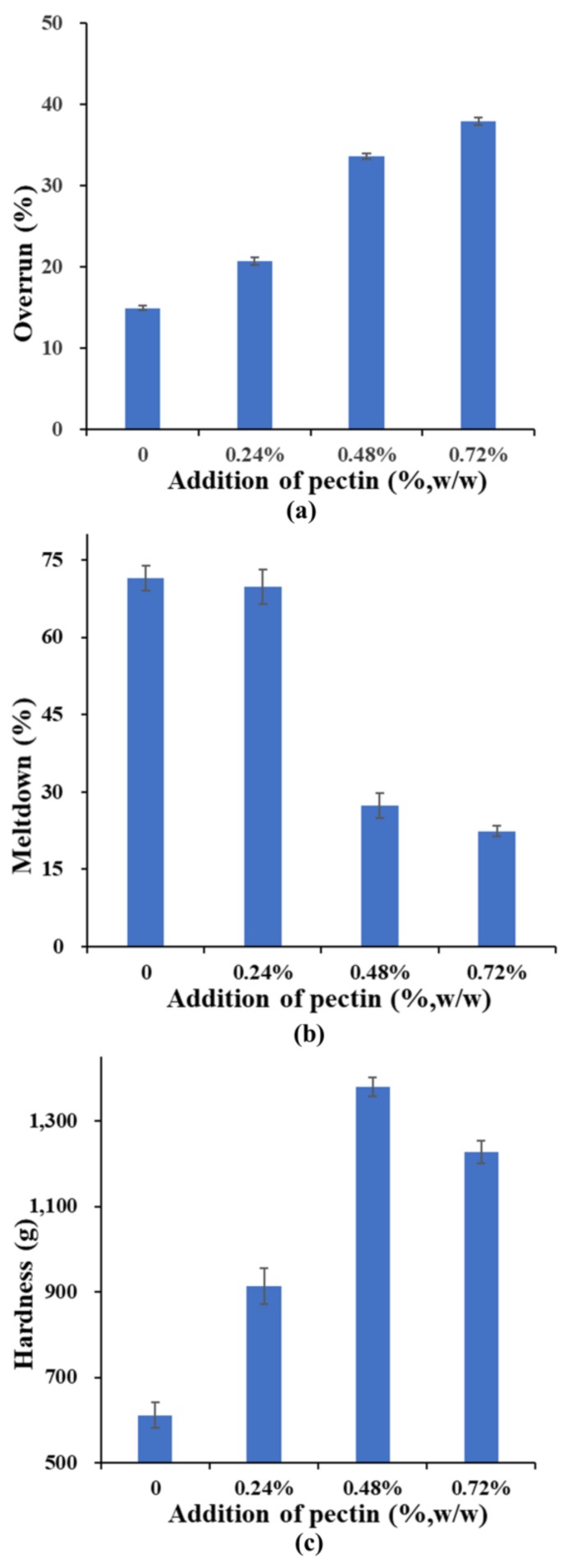
The influence of pectin addition on the (**a**) overrun, (**b**) meltdown and (**c**) hardness of ice cream.

The effects of pectin addition and fat reduction on the overrun (a), meltdown (b), and hardness (c) of the ice cream are shown in Figure 3. Many factors such as total solid contents, the viscosity of mix, the amount of air incorporated, volume and the size of ice crystals, and the fat globules were reported to affect the overrun, melting properties, and hardness of ice cream [11,22,23].

The statistical analysis results showed that the addition of pectin caused an increase in overrun of the ice cream, with a positive contribution to air incorporation. Incorporating air into ice cream mixes is an important step in the manufacture of ice cream, as it provides a soft texture to the products [24]. Pectin as a hydrophilic colloid can stabilize air bubbles so that air can be easily combined into ice cream in the process of whipping. In addition, the incorporation of pectin increased the viscosity of ice cream mix by enhancing gel structure in the liquid, which is beneficial because it stops air bubbles from coalescing [18]. 

A fast-melting product is unwanted while a too slow rate of melting can also be a defect of ice cream. As depicted in Figure 3b, the additions of pectin resulted in a decrease in the meltdown of the ice cream, and lead to greater product stability. Pectin can interact with other components in milk to a form dense three-dimensional network structure, which can slow down the flow of slurry and thus lower the heat transfer rate [19]. The addition of pectin also increased the viscosity of the slurry, which decreased the fluidity of the slurry and strengthened the melting resistance. Meanwhile, air is a poor conductor of heat energy and thus has negative effects on meltdown of ice cream with larger overrun [25].

Hardness is an important property of frozen products. On the one hand, ice cream must be hard enough so that it can be stored for a certain time and able to resist pressure. On the other hand, its hardness cannot be too high to affect the taste [18]. The results showed that the hardness of ice cream reached a maximum at a pectin concentration of 0.48%, and then falls off as the concentration increases. Compared to the control ice cream, the higher hardness of T1 (ice creams containing 0.24% pectin) and T2 (ice creams containing 0.48% pectin) ascribe to their lower fat content, higher viscosity and larger air cells resulting from higher overrun [23]. A similar trend was observed when xanthan, guar, carboxyl methyl cellulose (CMC) and sodium alginate were incorporated into ice cream [20]. T3 (ice creams containing 0.24% pectin) had lower hardness than T2 may due to that T3 had higher air bubble contents. 

### 2.5. Dynamic-Viscoelasticity of Ice Cream

**Figure 4:**
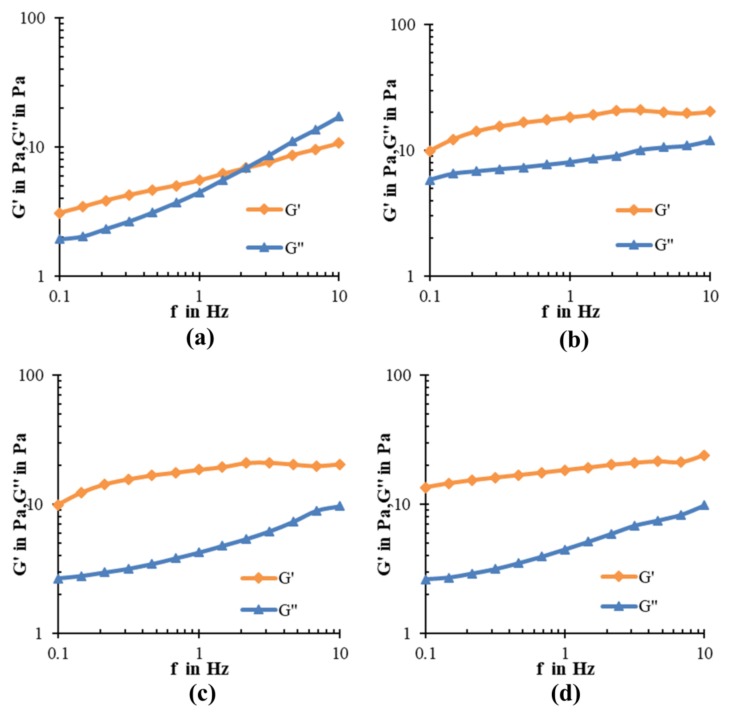
Frequency sweep of (**a**) Control (**b**) T1 (**c**) T2 (**d**) T3 ice cream at a strain of 0.01%.

To find the linear viscoelastic region of ice cream samples, the amplitude sweep (*γ* = 0.001–10%) at a fixed angular frequency (1 Hz) was conducted (Figure S1). Based on the strain amplitude sweep results, the frequency sweep test was performed at 0.01% strain (Figure 4). The test can simulate fluid behavior of the ice cream samples during mouth chewing, which is conducive to comprehensively evaluate the influence of pectin addition on the quality of ice cream. In the measurements, the elasticity can be described by the storage shear modulus, G′, and the viscous property can be described by the loss shear modulus, G″ [26]. The results showed that G′ and G″ of all samples increased with the frequency. This frequency dependence behavior of G′ and G″ is an indication of a typical gel behavior [27]. In the control samples, G′ was greater than G″ at frequencies 0.1–2 Hz, but, from 2 Hz onwards, the G″ was higher than G′, which means transition from more elastic behavior (G″ < G′) to more viscous behavior (G″ > G′) occurred. The results also indicated that G″ increased at a faster rate than G′. For the other three samples, G′ was greater than G″ throughout the frequency range tested (0.1 to 10 Hz) without any crossover points. This frequency dependence of the G′ and G″ allows these samples to be categorized as “physical gels” [28,29]. The values of G′ and G″ for control mixes were lower than pectin added mixes which indicated that the total modulus was enhanced with pectin. This could be attributed to the effect of entanglement network between gum and milk constituents [30]. 

### 2.6. Sensory Properties of Ice Creams

The results of the sensory evaluation of the ice cream samples are listed in Table 3. The appearance of the samples was not significantly different. The flavor and taste of T1 and T3 samples do not show significant difference compared to the control samples. T2 samples have relative lower flavor and taste scores than control samples. Moreover, the score of mouthcoating of T2 and T3 is lower than that of the control, which may ascribe to the higher viscosity of T2 and T3 samples. A smooth texture is important for the quality of ice cream product and an indicator of uniform texture and no detectable crystals [31]. The scores of smoothness were significantly improved with the addition of pectin, which is reasonable as pectin can be used as a stabilizer in ice cream. Stabilizers in ice cream provide several beneficial effects and one of the primary is producing smoothness in body and texture. 

## 3. Materials and Methods

### 3.1. Extraction of Pectin

**Figure 5:**
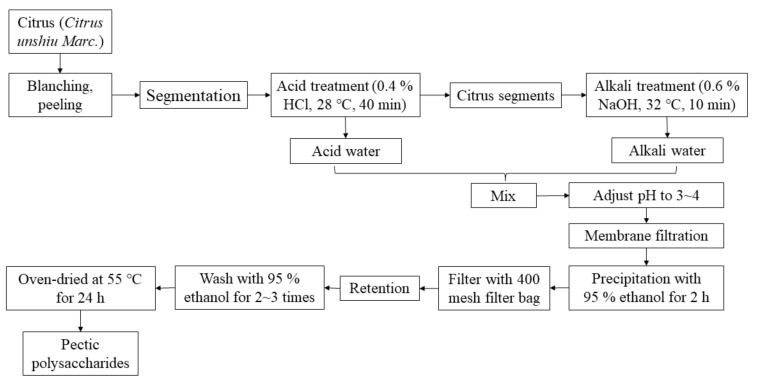
Extraction procedures of pectin.

The extraction procedures of pectin are showed in Figure 5. Acid and alkali wastewater were originated from removing segments membranes in a factory in Zhejiang province, China. First, the pH of mixtures containing acid and alkali waste water were adjusted to the ranges of 3 to 4. Then membrane filtration was applied to obtain the concentration pectin liquids. After that, 95% edible alcohol was added in the volume ratio of 1:1 to precipitate pectic polysaccharides. The obtained pectic polysaccharides were then washed by the 95% alcohol for 2–3 times and oven-dried at 55 °C for 24 h to obtain the dried pectin.

### 3.2. Monosaccharide Analysis of Pectin

Monosaccharide composition was analyzed by a 1-phenyl-3-methyl-5-pyrazolone (PMP) high-performance liquid chromatography (HPLC) method according to our previous study [2]. Briefly, pectic polysaccharides samples were first hydrolyzed with trifluoroacetic acid at 110 °C for 8 h, after which the acid was removed using a stream of nitrogen and neutralized with sodium hydroxide. The dried hydrolyzates were dissolved in sodium hydroxide and derivatized using PMP solution at 70 °C for 30 min. Finally, the mixtures were neutralized by hydrochloric acid, and chloroform was used to extract excess reagent. The upper phase was filtered through a 0.22 μm membrane and 1 mL of the resulting solution was injected for analysis. 

Waters e2695 (Waters, Milford, MA, USA) with a Zorbax Eclipse XDB-C18 column (250 mm × 4.6 mm, 5 μm, Agilent, Santa Clara, CA, USA) was used to perform HPLC analysis at 25 °C. The mobile phases were: solvent A, 15% acetonitrile with potassium phosphate buffer (0.05 M, pH 6.9), solvent B, 40% acetonitrile with the same buffer. The elution rate was 1 mL/min, relying on a gradient of B from 0% to 15% in the initial 10 min, then from 15% to 25% in the next 20 min. Detection was with a 2489 UV/Vis Detector (Waters, Milford, MA, USA) at 250 nm. 

Monosaccharide standards, PMP and D_2_O were all purchased from Sigma-Aldrich (Shanghai, China). All other chemicals used were of analytical grade.

### 3.3. Rheological Analysis of Pectin

Rheological measurements were carried out using a HAAKE RheoStress 6000 rheometer (Thermo Scientific, Waltham, MA, USA) with a P60 TiL parallel plate. The temperature was maintained at 25 ± 0.1 °C. To investigate the effects of concentration on rheological behavior of pectin, solutions of different concentrations including 0.5%, 1.0%, 1.5% (*w*/*v*) were prepared with deionized water and placed at room temperature for 24 h before use.

### 3.4. Preparation of Ice Cream

Skim milk powder (Nestle, Beijing, China), cream (35% fat) (Nestle, Beijing, China), pasteurized milk (Mengniu, Hohhot, China), sucrose and eggs were purchased from local suppliers. The ice cream formulations are listed in Table 4. The 2 kg batches of ice cream were prepared by mixing the ingredients according to Table 4. In addition, the pectins partially replaced the cream at level of 0 (control samples), 0.24 (T1), 0.48 (T2) and 0.72 (T3) (*w*/*w*). Egg yolks were weighed and whipped up. Milk powder, pectin and sucrose were weighed and dissolved at 60 °C for 30 min. The whipping components and solutions were then mixed, and water was made up according to the formulations. After that, the mixtures were pasteurized at 80 ± 1 °C for 30 s using a water bath plate. Then all mixtures were homogenized individually at 65 °C by a laboratory colloid mill (JM-L80, Taiya equipment company, China), and then cooled to 20 °C. Sterilized milk was added to the cooled samples to ensure the flavors. All mixtures were aged at 4 °C for 24 h to implement complete hydration of all ingredients. Subsequently, the mixtures were frozen in a batch ice cream freezer (Taylor161-40, Rockton, IL, USA) for 30 min. The final ice cream samples were filled into 100 mL plastic cups, hardened at −30 °C for 24 h and stored at −18 °C before further physical and chemical analysis. 

### 3.5. Overrun and Meltdown Test of Ice Creams

Overrun is usually defined as the volume of ice cream obtained in excess of the volume of the mix. It is usually expressed as “percent overrun”. It is one of the important parameters for determining the quality of ice cream. The overrun (%) of ice cream was determined by comparing the weight of the mixture before and after freezing, and calculated according to Equation (2) [32]:

(2) $$Overrun\%=\frac{weight\;of\;ice\;cream\;mix-weight\;of\;ice\;cream}{weight\;of\;ice\;cream}\times 100\%$$

Ice cream meltdown is one of the important manifestations of ice cream structure when the ice cream is at ambient conditions. Two factors related to the meltdown are melting of the ice and the collapse of the fat-stabilized foam structure. The meltdown is usually expressed as “percentage” and can be determined following the procedure below. 

The ice cream initially stored at −18 °C was cut by a cooled knife into a standard block (2 cm × 2 cm × 2 cm) to measure the meltdown. The standard block was then placed on a mesh in an enclosed chamber (DHG-101-156, Lantian, China) that was held at 25 ± 0.1 °C and allowed to melt. A funnel is placed beneath the mesh to collect the melted ice cream, which drains into a beaker on a balance. The mass of melted ice cream is recorded after 40 min, and the melting rate is calculated according to Equation (3).

(3) $$Meltdown\left(\%\right)=\frac{weight\;of\;the\;melted\;ice\;cream}{weight\;of\;the\;ice\;cream\;before\;melt}\times 100\%$$

### 3.6. Viscosity Determination of the Ice Cream Mixtures

Viscosity of the ice cream mixtures before freezing was measured by HAAKE RheoStress 6000 rheometer (Thermo Scientific, Waltham, MA, USA) with a P60 TiL parallel plate. The temperature was maintained at 25 ± 0.1 °C. The shear rates were changed from 0.1 to 100 s^−1^.

### 3.7. Hardness Determination of Ice Creams

The hardness of the ice cream was measured with a TA.XT2i texture analyzer (Stable Micro Systems Ltd., Godalming, UK). The test conditions were: temperature (4 °C), cylinder stainless probe (P/5), diameter 5 mm, pre-test speed 8 mm/s, test speed 2 mm/s, post-test speed 8 mm/s, depth of penetration 15 mm, and each sample was measured in triplicate.

### 3.8. Dynamic-Viscoelastic Determination of Ice Creams

The dynamic-viscoelastic properties of ice cream by using small amplitude oscillatory shear tests were performed on the hardened ice cream. The linear viscoelastic ranges were determined by amplitude sweep from 0.001% to 100% at a constant angular frequency of 1 Hz. The angular frequency sweep was conducted from 0.1 to 10 Hz at 0.01% deformation (smaller than the maximum value of linear viscoelastic range) to monitor the changing of storage modulus (G′) and loss modulus (G″) of the samples.

### 3.9. Sensory Evaluation 

A panel of 50 judges from our department at the Sensory Laboratory was invited to carry out the sensory evaluations on ice cream samples. They were selected based on regular consumption of ice cream and time availability. The training session and evaluation process were conducted in individual booths at the Sensory Laboratory under controlled lighting and temperature (25 °C). All samples were coded with random three-digit numbers and the serving orders were also randomized. A structured 9-point hedonic scale was employed for the evaluation of appearance, taste, flavor, smoothness, and mouth-coating of ice cream samples. The samples were rated according to the following criteria: nine for liked extremely, eight liked very much, seven liked moderately, six liked slightly, five neither liked nor disliked, four disliked slightly, three disliked moderately, two disliked very much and one disliked extremely.

### 3.10. Statistical Analysis 

All experiments were performed in triplicate and the statistical results were presented the average of three independent experiments. Statistical analyses were carried out by using the Statistical Analysis Systems (OriginPro 9.1, OriginLab Corporation, Northampton, MA, USA) software package. The results were evaluated by analysis of variance (ANOVA) and Duncan’s multiple range test at significance level of 0.05.

## 4. Conclusions

The present study showed that canned citrus wastewater can be an alternative source for production of pectin with the potential application as fat replacer for high-fat ice cream. First, the study confirmed that the pectin solutions are typical pseudoplastic fluids. Pectin recovered from canned citrus wastewater can be successfully incorporated into the ice cream to replace part of the fat. In addition, compared to the control ice cream, substitution of fat by pectin increased viscosity, overrun and hardness and reduced meltdown of ice cream. Sensory results indicated that pectin can replace 45% of the total fat of ice cream without changing its appearance, flavor, and taste scores. In addition, the low-fat ice cream had significantly higher smoothness score. 

## Figures and Tables

**Table 1:** Parameters of pectin flow curves obtained by fitting to power law model.

Concentration of Pectin	K (Pa·s^n^)	*n*	*R* ^2^
0.5%	0.11 ± 0.02	0.43 ± 0.01	0.99 ± 0.01
1.0%	0.40 ± 0.01	0.42 ± 0.01	0.99 ± 0.00
1.5%	1.46 ± 0.01	0.36 ± 0.01	0.98 ± 0.00

K: consistency index; *n*: flow behavior index; *R*^2^: model fitting degree.

**Table 2:** The values of rheological parameters of ice cream mixes.

Ice Cream Samples	K (Pa·s^n^)	*n*	*R* ^2^
Control	0.12 ± 0.01	0.79 ± 0.01	0.97 ± 0.01
T1	0.62 ± 0.01	0.45 ± 0.00	0.98 ± 0.00
T2	1.05 ± 0.02	0.37 ± 0.01	0.99 ± 0.00
T3	5.06 ± 0.02	0.25 ± 0.01	0.99 ± 0.00

**Table 3:** The score of each attributes of ice cream.

Ice Cream	Appearance	Flavor	Taste	Smoothness	Mouth Coating
Control	8.10 ± 1.02 ^a^	6.13 ± 1.23 ^a,b^	7.89 ± 1.57 ^a^	5.56 ± 2.05 ^b^	8.33 ± 0.97 ^a^
T1	7.22 ± 1.35 ^a^	6.56 ± 1.62 ^a^	7.78 ± 1.22 ^a^	7.56 ± 1.50 ^a^	8.00 ± 1.24 ^a^
T2	7.56 ± 1.67 ^a^	5.44 ± 1.49 ^b^	5.44 ± 2.00 ^b^	8.01 ± 1.23 ^a^	6.11 ± 1.84 ^b^
T3	7.24 ± 1.53 ^a^	6.33 ± 1.81 ^a,b^	7.11 ± 1.88 ^a^	7.46 ± 1.79 ^a^	6.77 ± 2.05 ^b^

^a^ and ^b^: means in the same row with different alphabets are significantly different (*p* < 0.05) for each attribute.

**Table 4:** Formulation of ice cream mixes (%, *w*/*w*).

Compositions	Treatments			
	Control	T1	T2	T3
Skim milk powder	20.00	20.00	20.00	20.00
Cream	15.00	12.75	10.50	8.25
Sucrose	10.00	10.00	10.00	10.00
Egg	1.60	1.60	1.60	1.60
Sterilized milk	5.00	5.00	5.00	5.00
Water	48.40	50.41	52.42	54.43
Pectin	0	0.24	0.48	0.72

## Supplementary Materials

The following are available online. Figure S1: The G′ (storage shear modulus) and G″ (loss shear modulus) of Control (a), T1 (b), T2 (c), T3 (d) ice cream from strain amplitude sweep (*γ* = 0.001–10%) at a fixed angular frequency (1 Hz), Table S1: The chemical composition of ice cream.

## References

[1] Wu D., Cao Y., Chen J., Gao H., Ye X., Liu D., Chen S. (2015). Feasibility study on water reclamation from the sorting/grading operation in mandarin orange canning production. J. Clean. Prod..

[2] Chen J., Cheng H., Wu D., Linhardt R.J., Zhi Z., Yan L., Chen S., Ye X. (2016). Green recovery of pectic polysaccharides from citrus canning processing water. J. Clean. Prod..

[3] Pagliaro M., Ciriminna R., Chavarría-Hernández N. (2015). Pectin: A new perspective from the biorefi nery standpoint. Biofuels Bioprod. Biorefin..

[4] Lim J., Ko S., Lee S. (2014). Use of yuja (*Citrus junos*) pectin as a fat replacer in baked foods. Food Sci. Biotechnol..

[5] Min B.K., Inyoung B., Hyeongyu L., Sangho Y., Suyong L. (2010). Utilization of pectin-enriched materials from apple pomace as a fat replacer in a model food system. Bioresour. Technol..

[6] Zaidel A., Hamidon N.H., Zahir N.M. (2017). Extraction and characterization of pectin from sweet potato (ipomoea batatas) peels using alkaline extraction method. Acta Hortic..

[7] Oliveira T.Í.S., Rosa M.F., Cavalcante F.L., Pereira P.H.F., Moates G.K., Wellner N., Mazzetto S.E., Waldron K.W., Azeredo H.M.C. (2016). Optimization of pectin extraction from banana peels with citric acid by using response surface methodology. Food Chem..

[8] Kang J., Hua X., Yang R., Chen Y., Yang H. (2015). Characterization of natural low-methoxyl pectin from sunflower head extracted by sodium citrate and purified by ultrafiltration. Food Chem..

[9] Prentice J.H. (1992). Dairy Rheology: A Concise Guide.

[10] Mcghee C.E., Jones J.O., Park Y.W. (2015). Evaluation of textural and sensory characteristics of three types of low-fat goat milk ice cream. Small Rumin. Res..

[11] Akalın A.S., Karagözlü C., Ünal G. (2008). Rheological properties of reduced-fat and low-fat ice cream containing whey protein isolate and inulin. Eur. Food Res. Technol..

[12] Lim J., Inglett G.E., Lee S. (2015). Response to consumer demand for reduced-fat foods; multi-functional fat replacers. Jpn. J. Food Eng..

[13] Methacanon P., Krongsin J., Gamonpilas C. (2014). Pomelo (*Citrus maxima*) pectin: Effects of extraction parameters and its properties. Food Hydrocoll..

[14] Velasco S.E., Areizaga J., Irastorza A., Dueñas M.T., Santamaria A., Muñoz M.E. (2009). Chemical and rheological properties of the beta-glucan produced by Pediococcus parvulus 2.6. J. Agric. Food Chem..

[15] Samanta A., Bera A., Ojha K., Mandal A. (2010). Effects of alkali, salts, and surfactant on rheological behavior of partially hydrolyzed polyacrylamide solutions. J. Chem. Eng. Data.

[16] Xu J., Inglett G.E., Chen D., Liu S.X. (2013). Viscoelastic properties of oat β-glucan-rich aqueous dispersions. Food Chem..

[17] Nascimento G.E., Simas-Tosin F.F., Iacomini M., Gorin P.A., Cordeiro L.M. (2016). Rheological behavior of high methoxyl pectin from the pulp of tamarillo fruit (*Solanum betaceum*). Carbohydr. Polym..

[18] Clarke C. (2004). The Science of Ice Cream.

[19] Varela P., Pintor A., Fiszman S. (2014). How hydrocolloids affect the temporal oral perception of ice cream. Food Hydrocoll..

[20] Soukoulis C., Chandrinos I., Tzia C. (2008). Study of the functionality of selected hydrocolloids and their blends with κ-carrageenan on storage quality of vanilla ice cream. LWT Food Sci. Technol..

[21] Oyaberkay K., Mehmet G., Kurban Y., Sevim K., Talip K. (2010). The functional, rheological and sensory characteristics of ice creams with various fat replacers. Int. J. Dairy Technol..

[22] Javidi F., Razavi S.M.A., Behrouzian F., Alghooneh A. (2016). The influence of basil seed gum, guar gum and their blend on the rheological, physical and sensory properties of low fat ice cream. Food Hydrocoll..

[23] Muse M.R., Hartel R.W. (2004). Ice cream structural elements that affect melting rate and hardness. J. Dairy Sci..

[24] Balthazar C.F., Silva H.L., Celeguini R.M., Santos R., Pastore G.M., Junior C.A., Freitas M.Q., Nogueira L.C., Silva M.C., Cruz A.G. (2015). Effect of galactooligosaccharide addition on the physical, optical, and sensory acceptance of vanilla ice cream. J. Dairy Sci..

[25] Sofjan R.P., Hartel R.W. (2004). Effects of overrun on structural and physical characteristics of ice cream. Int. Dairy J..

[26] Wildmoser H., Scheiwiller J., Windhab E.J. (2004). Impact of disperse microstructure on rheology and quality aspects of ice cream. LWT Food Sci. Technol..

[27] Kurt A., Kahyaoglu T. (2015). Rheological properties and structural characterization of salep improved by ethanol treatment. Carbohydr. Polym..

[28] Geresh S., Adin I., Yarmolinsky E., Karpasas M. (2002). Characterization of the extracellular polysaccharide of *Porphyridium* sp.: Molecular weight determination and rheological properties. Carbohydr. Polym..

[29] Xu J.L., Zhang J.C., Liu Y., Sun H.J., Wang J.H. (2016). Rheological properties of a polysaccharide from floral mushrooms cultivated in Huangshan Mountain. Carbohydr. Polym..

[30] Kurt A., Cengiz A., Kahyaoglu T. (2016). The effect of gum tragacanth on the rheological properties of salep based ice cream mix. Carbohydr. Polym..

[31] Bahramparvar M., Tehrani M.M., Razavi S.M.A. (2013). Effects of a novel stabilizer blend and presence of κ-carrageenan on some properties of vanilla ice cream during storage. Food Biosci..

[32] Tiwari A., Sharma H.K., Kumar N., Kaur M. (2015). The effect of inulin as a fat replacer on the quality of low-fat ice cream. Int. J. Dairy Technol..

