# Supplementary material for: Pectin from Citrus Canning Wastewater as Potential Fat Replacer in Ice Cream
Source: Molecules. 2018 Apr 17;23(4):925. doi: 10.3390/molecules23040925 (PMC6017722; doi:10.3390/molecules23040925)
Supplement: Supplementary file 1 [file molecules-23-00925-s001.pdf]

Supplementary Materials

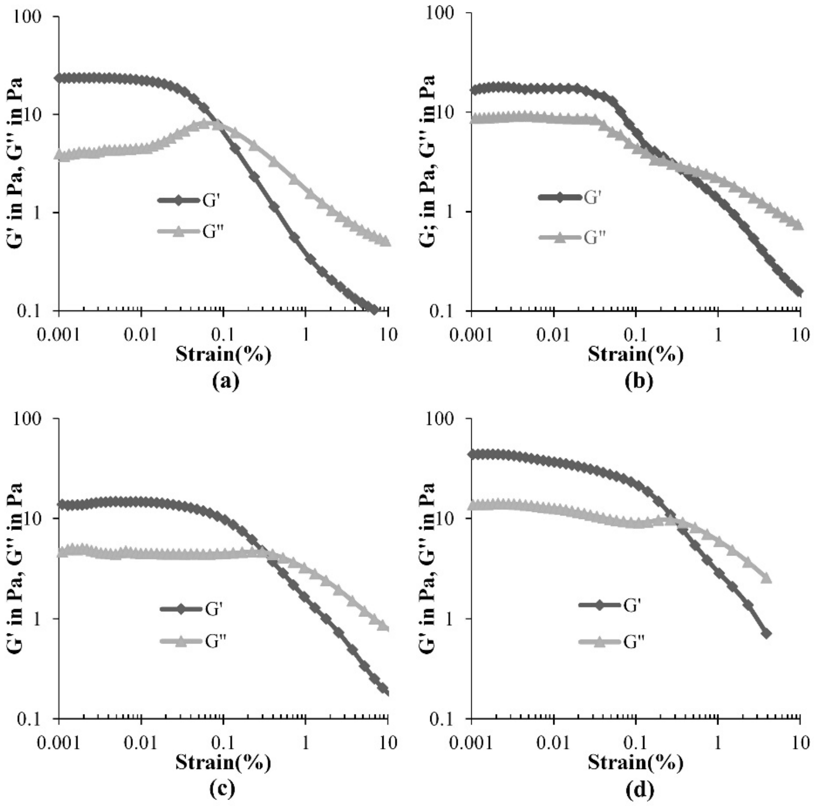

Figure S1. The G'(storage shear modulus) and G''(loss shear modulus) of Control(a) ,T1 (b),T2(c), T3(d) ice creams from strain amplitude sweep (γ= 0.001%-10%) at a fixed angular frequency (1Hz).
